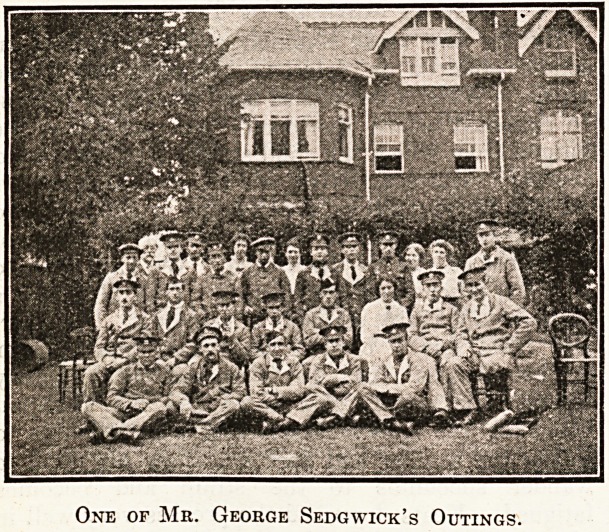# Personal Service in Leicester: Some Fruits of War-Time

**Published:** 1915-11-06

**Authors:** 


### PERSONAL SERVICE IN LEICESTER.

### Some Fruits of War-Time.

The record of personal service now being chronicled from all parts of the kingdom is possibly one of the most interesting and hopeful sidelights created by the war. It is interesting for it shows the Christian willingness of all classes to take a share in providing for others, and hopeful because the knowledge of distress and suffering gained thereby, and the possession of the desire to alleviate them, will carry a message into future years. The pessimist, in fact, will gain a most salutary lesson if he will but observe a portion of the willing service that is now being rendered freely and unostentatiously. And

One of Mr. George Sedgwick's Outings.
Back row, to the left, Mr. J. Leeson, the host of the day.

what is true of individuals is also true of communities. From Leicester we have recently learned of a series of systematic organisations which must inevitably arouse an interest that will last long after the war is over. The Royal Infirmary Matinée Committee, a body of well-known Leicester men who recognise the value of personal service, recently organised a special matinée at the local Palace Theatre to 2,000 wounded soldiers. This was made possible by a generous offer from Mr. Oswald Stoll to place the theatre, staff, artistes, and orchestra at the disposal of the Matinée Committee for the purpose. This committee has been the means of raising some £1,200 for the Leicester Royal Infirmary and local charities. This effort for the wounded is evidence of zeal and enthusiasm, and will enable the reader to appreciate the powerful inspiration it was to observe the merriment of 2,000 wounded soldiers.

But from the public point of view, which we have immediately in mind, the conveyance of the wounded from the hospitals to the theatre must inevitably inspire help in the relief of sickness, which will, we hope, create a new service having an important bearing upon hospital work in the years to come. Mr. George Sedgwick, a member of the board of the infirmary, was one of the first to put into operation this new method of service to the sick in Leicester. Twice a week throughout the summer he has secured the co-operation of his motoring friends to take parties of wounded soldiers under treatment at the infirmary for country drives, and well-known county residents have readily responded to the invitation to entertain the parties to tea and recreation. The members of the Leicester Automobile Club have also rendered systematic service in the same direction, and have on a large scale afforded opportunities to hundreds of soldiers, complete strangers to the town, to enjoy the surrounding picturesque scenery. Such usefulness is a worthy example of the utilisation of spare time for the welfare of others. It has brought the work of the Leicester hospitals prominently to the notice of residents in town and country. We cannot too strongly impress upon all hospital administrators the desirability of keeping in close touch with those who have rendered yeoman service in this direction, and effectively to apply to the welfare of their institutions the interest which has thus been created. We cannot doubt that unselfish service, which has been of such great gain to wounded soldiers, will also be organised as a supplemental service in the restoration to health of civil patients in the voluntary hospitals. Personal service of this kind broadens the outlook of those who render it, and has an educative influence on the whole community of the utmost value.

## Figures and Tables

**Figure f1:**